# Brain natriuretic peptide is related to diastolic dysfunction whereas urinary albumin excretion rate is related to left ventricular mass in asymptomatic type 2 diabetes patients

**DOI:** 10.1186/1475-2840-9-2

**Published:** 2010-01-18

**Authors:** Martin Magnusson, Stefan Jovinge, Kambiz Shahgaldi, Bo Israelsson, Leif Groop, Olle Melander

**Affiliations:** 1 Department of Cardiology, Malmö University Hospital, Lund University, Lund, Sweden; 2 Department of Cardiology, Lund University Hospital, Lund University, Lund, Sweden; 3 Lund Strategic Research Centre for Stem Cell Biology and Cell Therapy, Lund University, Lund, Sweden; 4 Department of Cardiology, Karoliniska University Hospital, Stockholm University, Sweden; 5 Lund University Diabetes Centre, Clinical Research Centre, Malmö University Hospital, Lund University, Sweden

## Abstract

**Background:**

The aims of this study were to estimate the prevalence of left ventricular systolic (LVSD) and diastolic (LVDD) dysfunction, and to test if BNP and urinary albumin excretion rate (AER) are related to LVSD, LVD and left ventricular mass (LVM) in asymptomatic type 2 diabetes patients.

**Methods:**

Presence of LVSD, LVDD and LVM, determined with echocardiography, was related to levels of BNP and AER in 153 consecutive asymptomatic patients with type 2 diabetes.

**Results:**

LVSD was present in 6.1% of patients whereas 49% (29% mild, 19% moderate and 0.7% severe) had LVDD and 9.4% had left ventricular hypertrophy. Increasing age (P < 0.0001) was the only independent variable related to mild LVDD whereas increasing BNP (P = 0.01), systolic blood pressure (P = 0.01), age (P = 0.003) and female gender (P = 0.04) were independent determinants of moderate to severe LVDD. AER (P = 0.003), age (P = 0.01) and male gender (P = 0.006) were directly and independently related to LVM.

**Conclusion:**

About half of asymptomatic type 2 diabetes patients have LVDD. Of those, more than one third display moderate LVDD pattern paralleled by increases in BNP, suggesting markedly increased risk of heart failure, especially in females, whereas AER and male sex are related to LVM.

## Background

The steadily increasing prevalence of diabetes constitutes a major health problem. By the year 2025, the prevalence of diabetes is estimated to be as high as 5.4% [1]. This is to be compared with reports from Swedish coronary care units, which suggest that as many as 66% of the patients admitted in fact have previously undiagnosed diabetes or pre-diabetes (impaired glucose tolerance) [2]. Cardiovascular disease (CVD) is also the major contributor to mortality in the diabetic population [3]. Screening for micro-vascular complications has been routine for decades, but no similar practice for early detection and treatment of cardiac complications has been conducted. This is especially notable, since treatment at different stages of heart dysfunction, even asymptomatic, has proved to reduce cardiovascular complications [4,5]. It is also well known that microalbuminuria is a powerful predictor of CVD, especially in patients with type 2 diabetes [6], and this subset group would probably benefit even more from a cardiac screening. Routine echocardiography (echo) assessment would be considered optimal for such conduct, but is not always possible due to lack of accessibility. The measurement of the natriuretic peptides has been suggested as a tool to identify patients who could benefit from further risk evaluation by echo for the early detection and treatment of cardiac abnormalities [7]. We have previously reported that, in a material of patients with type 2 diabetes without known cardiovascular disease, over 60% of the patients had N-terminal proBNP values above accepted cut off limits for the detection of cardiac abnormalities [8]. However, no echo examinations were conducted in that study and it is unknown whether clinically easy-to-use markers like BNP and microalbuminuria can be used to identify type 2 diabetes patients with early signs of heart disease such as left ventricular dysfunction (LVD) and left ventricular hypertrophy (LVH). Therefore, in this study we set out to estimate the prevalence of left ventricular systolic dysfunction (LVSD), left ventricular diastolic dysfunction (LVDD) and LVH as measured by echo in a population of patients with type 2 diabetes and no history or symptoms of CVD, and further, in the same population, to test if BNP and urinary albumin excretion rate (AER) are related to LVSD, LVDD and left ventricular mass index (LVM) independently of each other, age, sex, diabetes duration, systolic blood pressure, metabolic control, lipid levels, body mass index and kidney function.

## Materials and methods

### Patients and protocol

The study population consisted of 153 consecutive patients with type 2 diabetes with no history of symptoms of cardiovascular disease being part of a screening program for heart disease in type 2 diabetes patients without known macrovascular disease in Malmö, Sweden. This is a joint venture between the primary care in Malmö and the departments of cardiology and endocrinology at Malmö University Hospital. Patients with newly diagnosed diabetes without known cardiovascular disease are examined thoroughly, (e.g. with echo and a broad range of cardiovascular risk factors) and interventions are executed on the basis of the examination results. The patients were admitted consecutively and the mean age was 55 ± 11 years, though our aim was to examine newly diagnosed diabetes patients, the duration of diabetes was found to be longer than expected, 6.3 ± 7.2 years and 46% of the patients had hypertension. Clinical and biochemical characteristics are shown in table 1 and echo variables are shown in table 2. The local ethics committee of Lund University approved the study, which was conducted according to the principles of the Declaration of Helsinki and thus all participating patients gave their written informed consent and procedures involving patients were performed according to institutional guidelines.

**Table 1:** Clinical characteristics of the study population.

Variable	Patients without LVDD n = 78 Mean	Patients with LVDD n = 75 Mean	p value
Age	50 ± 12	59 ± 10	< 0.001
Sex (% male)	51	49	0,1
SBP (mmHg)	134 ± 16	141 ± 16	0.006
DBP (mmHg)	79 ± 9.0	80 ± 8.0	0.6
HT (%)	36	58	0.009
Crea ((μmol/L)	73 ± 19	81 ± 42	0.1
GFR (ml/min)	137 ± 52	116 ± 41	0.005
Dyslipidemia (%)	51	49	0,3
lnAER ( g/min)	2.1 ± 1.3	2.3 ± 1.7	0.4
lnBNP (pmol/L)	1.1 ± 1.0	1.6 ± 1.1	0.01
HbA1c (%)	7.0 ± 2.0	6.5 ± 1.4	0.04
fP-glucose (mmol/L)	9.3 ± 3.8	8.9 ± 3.0	0.5
DM duration (years)	4,7 ± 6,6	8.0 ± 7.6	0.005
Smoking (%)	15	9	0.2
BMI (kg/m^2^)	30 ± 6.1	31 ± 5.2	0.3
TG (mmol/L)	1.8 ± 1.1	1.8 ± 1.0	1.0
HDL (mmol/L)	1.1 ± 0.3	1.2 ± 0.4	0.3
LDL (mmol/L)	2.9 ± 0.8	2.7 ± 1.0	0.2
BSA (m^2^)	2.0 ± 0.24	2.0 ± 0.20	0.5

Data are expressed as mean ± SD. SBP; systolic blood pressure, DBP; diastolic blood pressure, HT; hypertension, Crea; creatinine, GFR, glomerular filtration rate, AER, urinary albumin excretion rate, BNP, brain natriuretic peptide, f; fasting, DM; diabetes mellitus, BMI; body mass index, TG; triglycerides, HDL; high density lipoproteins, LDL; low density lipoproteins, BSA; body surface area, ln; natural logarithm.

**Table 2:** Echocardiographic parameters

Variable	Patients without LVDD n = 78 Mean	Patients with LVDD n = 75 Mean	p value
EF (%)	60 ± 6.2	56 ± 10	0.03
E (cm/s)	78 ± 15	79 ± 18	0.8
A (cm/s)	68 ± 15	97 ± 57	0.001
Edt (s)	0.2 ± 0.06	0.2 ± 0.05	0.2
Pulmonary vein flow	1.4 ± 0.7	1.2 ± 0.6	0.2
RA diameter (mm/m^2^)	24 ± 4,5	25 ± 4.4	0.3
IVSDd (mm/m^2^)	5.8 ± 1.0	6.0 ± 0.8	0.3
LVIDd (mm/m^2^)	24 ± 2.5	24 ± 2.6	0.5
RVIDd (mm/m^2^)	14 ± 1.9	15 ± 2.2	0.2
PWDd (mm/m^2^)	4.6 ± 0.7	4.5 ± 0.7	0.6
PSVsept (cm/s)	9.2 ± 6.8	7.7 ± 1.6	0.1
Èsept (cm/s)	10 ± 6.7	6.7 ± 1.8	0.001
Ásept (cm/s)	9.3 ± 2.0	10 ± 2.6	0.06
PSVlat (cm/s)	8.9 ± 2.4	8.4 ± 2.0	0.2
Élat (cm/s)	11 ± 3.2	9.1 ± 2.3	< 0.001
Álat (cm/s)	10 ± 3.6	11 ± 3.1	0.2
LA diameter (mm/m^2^)	20 ± 2.3	20 ± 1.9	0.7
LVM (g/m^2)^	87 ± 22	88 ± 25	0.9

Data are expressed as mean ± SD. BSA; body surface area, EF; ejection fraction, E; peak early mitral valve velocity, A; peak late atrial mitral valve velocity, Edt; deceleration time, LA; left atrium, RA; right atrium, IVSDd; interventricular systolic diameter diastole, LVIDd; left ventricular inner diameter diastole, RVIDd; right ventricular inner diameter diastole, PWDd; posterior wall diameter diastole, PSVsep; peak systolic myocardial velocity at mitral annulus in septal wall, Ésept; early diastolic tissue velocity at mitral annulus in septal wall, Ásept; late diastolic tissue velocity at mitral annulus in septal wall, PSVlat; peak systolic myocardial velocity at mitral annulus in lateral wall, Élat; early diastolic tissue velocity at mitral annulus in lateral wall, Álat; late diastolic tissue velocity at mitral annulus in lateral wall, LVM; left ventricular mass.

### Blood pressure

Blood pressure was measured three times in the right arm in seated position after 10 min rest and the mean value of the three recordings was calculated. Korotkoff sounds corresponding to 'phase I' was used to define the systolic and 'phase V' the diastolic blood pressure. Hypertension was defined as systolic or diastolic blood pressure of 140/90 mmHg or greater or use of antihypertensive medication.

### Diabetes

Diabetes mellitus was defined as a fasting whole blood glucose level greater than 109 mg/dL (6.0 mmol/L) or use of antidiabetic medication.

### Echocardiography

All patients underwent complete 2-dimensional echocardiography and Doppler studies in the left lateral decubitus position from multipline windows, using commercially available equipment (SONOS 7500, Philips Medical Systems, Andover, Massachusetts) with a phased array transducer of 2.5 MHz, and with a system equipped with Doppler tissue imaging technology. Pulsed Doppler tissue imaging is a new echo method based on the display of low frequency, high amplitude Doppler signals originated in the myocardium. A detailed concept and technical aspects of this approach have previously been published [9,10]. All images were recorded during two cardiac cycles and were stored digitally and post processed.

In addition to pulsed Doppler tissue imaging, conventional echo was performed, including M-mode, 2-dimensional, pulsed and colour Doppler echocardiography. Right ventricular and left ventricular diameters, left and right atrium diameters, septal and posterior wall thickness were measured according to the recommendations of the American Society of Echocardiography, and left ventricular ejection fractions were calculated using the biplane Simpsons [11]. The severity of valve regurgitations and stenoses was defined according to the clinical practice guidelines at Malmö University Hospital and in agreement with ACC/AHA guidelines [12].

The assessment of the severity of the valvular stenoses and regurgitations was performed using a combination of qualitative and quantitative analysis based on echocardiographic spectral and colour Doppler measurements.

**Figure 1:**
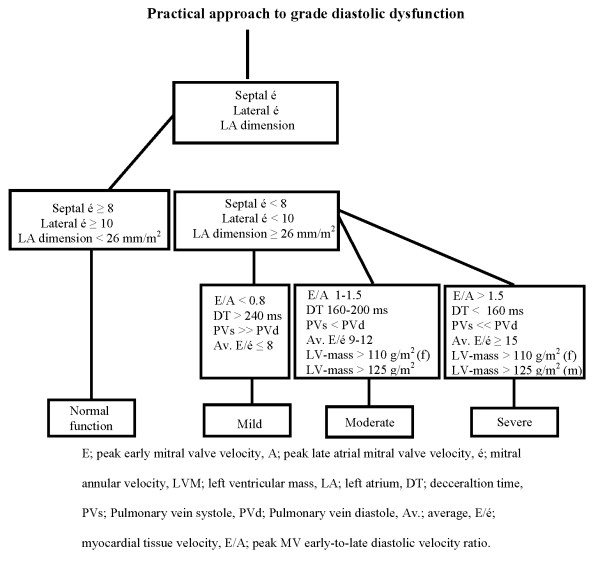
**Practical approach to grade diastolic dysfunction**.

The assessment of diastolic heart function was defined according to European Society of Cardiology [13] and LVDD was divided into mild (relaxation impairment), moderate (pseudonormalisation pattern) and severe (restrictive pattern). Diagnostic evidence of LVDD was obtained among other by tissue Doppler (E/é), blood flow Doppler of mitral valve, pulmonary veins and echo measures of LVM index (figure 1). LVSD was defined as ejection fraction below 55%. LVH was defined as LVM of at least 110 g/m^2 ^in women and 125 g/m^2 ^in men. LV-mass (LVM) was calculated according to the formula:

PWTd: posterior wall thickness in diastole, SWTd: septal wall thickness in diastole, LVIDd: left ventricular inner diameter in diastole.

### Biochemical assays

At the time of echocardiography, venous blood was sampled and transferred into pre-chilled EDTA vacutainers for analysis of BNP. Immediately after sampling the tubes were placed on ice and centrifuged at 4°C before plasma aliquots were frozen at -70°C for later analysis. Plasma BNP was measured with the Triage BNP Assay from Biosite Inc, San Diego, CA, using the UniCel™ immunoassay System from Beckman Coulter Inc, Brea, Ca. Lower limit of detection is 1 pg/mL (0.29 pmol/L). Within assay CVs are 3.1% and 1.0% at the levels of 40.8 and 1343 pg/mL. Corresponding between assay CVs are 4.5 and 6.6%. HbA1c was analysed using the Variant II chromatographic method from Bio-Rad (CA, USA) with a coefficient of variance (CV) of 3.0% at HbA1c 4.4-8.8%. The urinary albumin concentration was measured in over-night urine collections (12 hours) using an immunoturbimetric method (BeckmanCoulter, Beckman Instruments, CA, USA). Glomerular filtration rate (GFR) was calculated from the the Cockcroft-Gault formula; GFR (mL/min/1.73 m2) = ((140-age (years)) × body weight (kg))/(0.81 × S_creat _(mmol/l)) × (1:73 (m2)/BSA (m2))(×0.85 if female).

### Statistics

Normally distributed data are presented as means ± SD whereas data with skewed distributions are presented as medians and inter quartile ranges (IQR). Univariate differences in continuous variables was tested using Student's t-test with skewed variables being ln-transformed before analysis whereas chi-2 test was used to for differences in dichotomised variables. Correlation coefficients were calculated using Pearson's or Spearman's correlation analysis depending on normality of the residuals. Linear regressions, with skewed variables were ln transformed, and logistic regressions were used to test for multivariate relationships with continuous and dichotomous variables, respectively. All baseline variables (table 1) were entered into the multivariate models and were retained in the model if P < 0.05 using backward elimination. Two-sided tests were used and P < 0.05 was considered statistically significant throughout.

## Results

Clinical and biochemical characteristics are shown in table 1 and echo variables are shown in table 2. The mean blood pressure constitutes a mixture of blood pressure under treatment and untreated patients. On average patients had a LVM below the applied limits for LVH and 9.4% fulfilled the criteria for LVH. LVSD was only present in 6,1% of the subjects whereas 49% of the patients had echo signs of LVDD (29% mild, 19% moderate and 0.7% severe).

### BNP and AER in relation to LVM

In a univariate analyses lnAER was significantly correlated (r = 0.34, P < 0.0001) and lnBNP showed a strong tendency without reaching the level of statistical significance (r = 0.17, P = 0.06) with LVM. However, in a multivariate linear regression model including all baseline covariates (table 1), only AER (unstandardized β coefficient (β) and (±SD) = 4.3 ± 1.4, P = 0.003), age (β = 0.48 ± 0.18, P = 0.01) and male gender (β = -13 ± 4.7, P = 0.006) remained independently related to LVM.

### Correlations to diastolic dysfunction

When comparing patients with and without mild diastolic dysfunction (patients with moderate to severe LVDD excluded), we found no difference in lnBNP or lnAER, although there was a tendency towards higher lnBNP in patients with mild LVDD (1.3 ± 0.79 vs 0.98 ± 0.85, P = 0.09). Patients with mild LVDD were found to be significantly older (59 ± 7.6 vs 49 ± 11 years; P < 0.0001), have longer duration of diabetes (8.0 ± 8.0 vs. 4.6 ± 6.7 years; P = 0.02) and lower GFR (99 ± 27 vs 120 ± 33 ml/min/1.73 m^2^; P = 0.002). However, in a multivariate logistic regression analysis, increasing age (P < 0.0001) remained the only independent variable related to mild LVDD.

**Figure 2:**
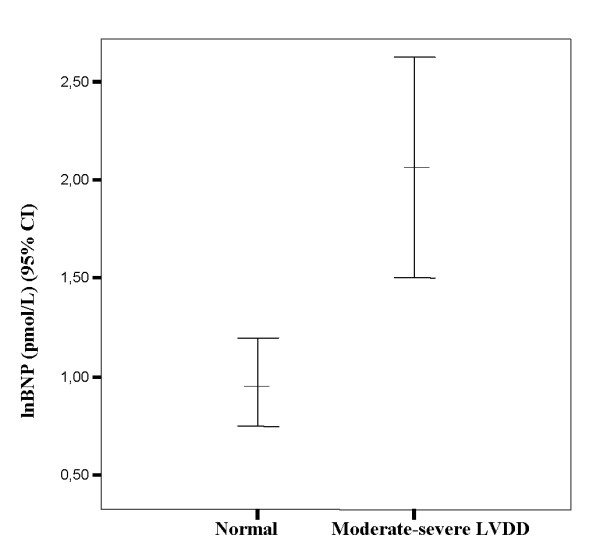
**Distribution of BNP values in the patient with normal and moderate-severe diastolic dysfunction**. P for difference between groups < 0.001.

Patients with moderate to severe LVDD had significantly higher lnBNP (2.1 ± 1.4 vs 0.98 ± 0.85 pmol/L; P < 0.0001) (figure 2) as compared to patients with normal left ventricular dysfunction, whereas there was no difference in AER (2.6 ± 2.0 vs 2.0 ± 1.3 μg/min; P = 0.12). In addition, they were significantly older (59 ± 13 vs 49 ± 11 years; P < 0.0001), had longer duration of diabetes (7.9 ± 7.2 vs 4.6 ± 6.7 years, P = 0.03), higher systolic blood pressure (146 ± 18 vs 133 ± 16 mmHg; P = 0.001) and lower GFR (97.0 ± 32.7 vs 118 ± 33.3 ml/min/1.73 m^2 ^P < 0.005). In multivariate logistic regression analysis the OR for moderate to severe LVDD per one unit increase in the respective variable was calculated. BNP, systolic blood pressure (SBP), age and female gender were all independently influencing the risk of moderate to severe LVDD per one unit increase in the respective variable. The multivariate adjusted OR (95% CI) for moderate to severe LVDD was 1.14 (1.03-1.25) for BNP, 1.05 (1.01-1.09) for SBP, 1.10 (1.03-1.16) for age and 3.61 (1.03-12.7) for female gender. Furthermore, a ROC analysis was performed to evaluate the diagnostic performance for BNP as a discriminator for the detection of moderate to severe LVDD, and was expressed as a calculation of area under curve (AUC) level. Regarding the ability to diagnose if moderate to severe LVDD was present, ROC analysis showed a significant diagnostic performance for BNP (AUC = 0.74 (0.62-0.85), p < 0.0001) for the detection of moderate to severe LVDD.

## Discussion

Although BNP is a well-established screening tool for LVSD and AER is routinely used for cardiovascular risk estimation, it is not known whether these two biomarkers are clinically useful for screening of LVDD and LVH in patients with type 2 diabetes with no prior CVD. The present study has demonstrated that echocardiographic screening of asymptomatic diabetic subjects without known CVD may identify significant numbers of patients with asymptomatic LVDD and LVH. We also show that BNP is independently related to moderate to severe LVDD whereas AER is directly and independently related to LVM.

### Prevalence of subclinical heart disease in a diabetic population

Our data shows a prevalence of 49% of LVDD (29% mild, 19% moderate and 0.7% severe LVDD) in presumably heart healthy asymptomatic patients with type 2 diabetes. These findings are well in line with earlier studies i.e. data from Poirier et al were 46 men with type 2 diabetes who were aged 38-67 years; without evidence of diabetic complications, hypertension, coronary artery disease, congestive heart failure, or thyroid or overt renal disease; and with a maximal treadmill exercise test showing no ischemia, were studied. Twenty-eight of the subjects (60%) were found to have LVDD, of whom 13 (28%) had moderate LVDD and 15 (32%) had mild LVDD [14].

In our study, LVH was observed in only 9.4% of the patients. Fang et al. reported that in asymptomatic patients with diabetes mellitus without known cardiac disease, which underwent clinical evaluation and detailed echo assessment, approximately 20% fulfilled the criteria for LVH.

It has recently been published data by Srivastava and coworkers, showing a high prevalence of LVDD and LVH (59% and 70% respectively) [15] in patients with type 2 diabetes. As in our study, the prevalence of LVSD was fairly low (16%). The prevalence of LVH reported in that study is higher compared to our findings (70% v s 9.4%). However in that study population, 19% had known macrovascular disease. Interestingly, increasing age, diabetes duration and BMI were the only independent predictors of cardiac abnormalities. No differences regarding cardiac abnormalities between male and females were reported for [15].

### BNP and correlations to echo abnormalities

We have previously reported that patients with type 2 diabetes have higher levels of BNP compared to patients without diabetes, indicating a high prevalence of asymptomatic heart disease [8]. However, since no echo registrations were done in this study [8], no information regarding what underlying heart disease an elevated BNP value would represent could be obtained. For screening purposes BNP have been proven useful to rule out heart failure (HF) with impaired LVSD [16]. BNP have also been shown useful for the assessment of LVDD at the population level [17] whereas its role for LVDD screening has not previously been investigated in type 2 diabetes patients with no prior CVD. We found that BNP is an independent determinant of moderate to severe LVDD. This is illustrated in figure 1 were BNP appears as good discriminator for the detection of moderate to severe LVDD in patients with type 2 diabetes. Henceforth, plasma BNP levels above 1.5 pmol/l indicates coexisting moderate diastolic dysfunction and further examination with echo would be warranted to further confirm this.

On the other hand, we did not found any significant relationship between BNP and mild LVDD or LVM, respectively. In accordance with the latter finding, screening with BNP for the presence of LVH in hypertensive patients has showed limited value [18].

### AER and correlations to echocardiographical abnormalities

Whereas BNP was not associated to LVH, our data suggests that AER could be useful as a marker for LVH, as estimated by LVM, given its independent and direct relationship with LVM. An independent relationship between LVM and AER has not been reported earlier in type 2 diabetes and further emphasizes increased CVD risk and need of antihypertensive treatment in type 2 diabetes patients with microalbuminuria. Thus, this finding might serve as an explanatory model for the known risk of CVD in patients with microalbuminuria [6], since LVH is well associated with increased morbidity and mortality in CVD [19].

### Male gender and left ventricular hypertrophy

The correlation between male gender and LVM is in line with the study by Concardy et al in 2004, in which it was shown that the LVH estimation without sex-specific criteria underestimated the prevalence of LVH in women and overestimates it in men, and even if sex specific definitions for LVH was used male gender still contributed to the prevalence of LVH in this study [20]. Furthermore, an independent influence of increasing LVM and age were reported by Concardy et al and this influence was only seen in the male population of the study. Even if the patients studied by Concardy et al were hypertensive peers with higher prevalence of LVH (52.2%) compared to our diabetic population with a LVH prevalence of 9.4%, the data are in concordance with our findings and might represent a gender specific mechanism for the development of LVH. This is further supported by data from the Tromsö study in which male sex was found to be an independent predictor of LVH in a multivariate logistic regression analysis [21].

### Female gender and diastolic dysfunction

Another interesting finding from the present study is the increased risk for moderate LVDD for female study subjects. The mechanism behind such a gender dependent pathophysiology is unclear. However, the results of the Framingham study revealed that between the ages of 45 and 75, men and women with diabetes had a two-fold- and five-fold increase in risk of developing heart failure, respectively, compared to patients without diabetes. This risk persisted even after considering age, blood pressure, cholesterol, weight, and history of coronary artery disease [22]. Further studies are indeed warranted regarding this matter.

### Screening for cardiac abnormalities in asymptomatic patients with type 2 diabetes

In our study almost half of the study population had LVDD and nearly ten percent had LVH. The treatment of LVH and LVSD is well established and has proved to reduce cardiovascular complication [4,5]. However, the importance of diagnosing LVDD has just been acknowledged and the presence of LVDD provide essential prognostic information [23]. Although the most effective treatment of LVDD in patients with type 2 diabetes is unknown, it is well established that an effective aggressive multifactorial approach indeed reduces micro- and macrovascular complications [24]. Furthermore, recently published data suggests cardiovascular benefits of more aggressive blood pressure lowering therapy amongst patients with type 2 diabetes [25]. Henceforth, an early detection of cardiac abnormalities such as LVSD, LVDD and LVH is crucial and might guide the clinicians to more intense risk factor management and aggressive pharmacological treatment of this high-risk population. Screening with echo would be considered optimal for such a conduct, but is not always eligible due to lack of accessibility and high cost. Our data suggest that the measurement of BNP and AER might be used as a screening tool in order to select patients for further work up with echo. However, the study sample size of a total of 153 subjects, which indeed is a respectable number for an echocardiographic study, might not be large enough to be definitive or inform clinical practice and therefore the results from the multivariate analysis needs to be confirmed in larger study samples.

## Conclusion

In conclusion, this study has demonstrated that echo screening of asymptomatic diabetic subjects with apparently normal cardiac function may identify significant numbers of patients with asymptomatic LVDD and LVH. Almost half of the presumably heart healthy population had LVDD. BNP discriminates patients at high risk for moderate to severe LVDD, and might therefore be used as screening tool in order to capture patients eligible for further risk stratification with echo. LVH was associated with male gender and AER, whereas moderate LVDD was associated with female gender and BNP, implying gender specific mechanism in the development of CVD.

## Competing interests

The authors declare that they have no competing interests.

## Authors' contributions

MM and OM have participated in the design of the study, performed the statistical analyses and drafted the paper. SJ, LG and BI conceived the study, participated in its design and coordination and helped to draft and review the manuscript. KS helped in the data organization and retrieval, English editing and final draft preparation. All of the authors have read and approved the final manuscript.
